# Editorial “Nutrition and Dietary Intake for Liver-Related Diseases”

**DOI:** 10.3390/nu13020390

**Published:** 2021-01-27

**Authors:** Ewa Stachowska, Karolina Jakubczyk, Dominika Maciejewska-Markiewicz

**Affiliations:** Department of Human Nutrition and Metabolomics, Pomeranian Medical University, 71-460 Szczecin, Poland; jakubczyk.kar@gmail.com (K.J.); dominika.maciejewska@pum.edu.pl (D.M.-M.)

In this special issue, we focus on the role of nutrition in the therapy of nonalcoholic liver disease (NAFLD). Currently, substantial evidence exists suggesting the important role played by intestinal microbiota in the pathogenesis of NAFLD. Functional connection of microbiota with the mucus layer, the enterocytes, and the gut immune system is crucial for intestinal permeability. Moreover, many dietary factors influencing the gut microbiota may lead to the elevation of intestinal permeability and increase the risk of NAFLD (as well as obesity and insulin resistance). It has been established that the presence of liver steatosis was associated with increased colonic permeability and lower adherence to the Mediterranean diet and a healthy lifestyle [1].

The meta-analysis by Stachowska and colleagues [2] showed that fiber supplementation can be related to favorable changes in body mass index (BMI), insulin homeostasis, and liver-related biomarkers, such as alanine aminotransferase (ALT) and aspartate aminotransferase, (AST) in NAFLD patients. NAFLD progression and liver cirrhosis are related to several conditions associated with decreased antioxidant capacities and increased oxidative stresses [3]. Lai et al. revealed that the severity of liver cirrhosis was associated with decreased glutathione and its related enzyme activity. However, a 12-week double-blind randomized controlled trial based on vitamin B6 and/or glutathione supplementation produced no significant effects on indicators of oxidative stress and antioxidant capacities [3].

Natural substances may exert antioxidant and anti-inflammatory effects and successfully support NAFLD therapy. Jakubczyk et al. performed a meta-analysis examining the effects of resveratrol supplementation in NAFLD patients. The study revealed no significant impact on reducing anthropometric parameters, lipid profile, glucose metabolism, or arterial pressure. The currently available evidence is insufficient to confirm the efficacy of resveratrol in the management of NAFLD, thus further research is needed to clarify this issue [4].

Inulin is a natural soluble fiber used as a food supplement and food additive improving nutritional value. Despite many reports on the health-promoting properties of dietary fiber, the effect of inulin on the liver seems unclear. Pauly et al. showed that relatively short-term inulin consumption in mice with an intact gut microbiome, disturbs cholesterol and bile acid metabolism and may have detrimental effects on liver function. Lowering of plasma cholesterol, induction of cholestasis, and mild liver injury were noted [5].

The gut microbiome is one of the important factors in chronic liver disease progression. Hussain et al. studied the association between diet and the microbiome of the proximal gut in patients with liver cirrhosis [6]. They confirm that increased protein, fiber, and coffee in the diet are associated with diversity and composition of the duodenal microbiome in liver cirrhosis [6].

Bile acids (BA) are synthesized in the liver from cholesterol and are important signaling molecules in a gut–liver axis. Primary BA undergo biotransformation to secondary BA by the intestinal microbiome and are reabsorbed into the portal tract of the liver. BA act as important signaling molecules of the nuclear farnesoid X receptor (FXR) and the membrane-associated G-protein-coupled bile acid receptor-1 (GPBAR-1) [1]. These receptors participate in metabolic pathways preventing the side effects of BA overload, including inflammation processes, thermogenesis, cholangiocyte secretion, biliary epithelial barrier permeability, enterohepatic circulation, and changes of epigenetic mechanisms [7].

## Data Availability

Not applicable.
